# Supplementary material for: Biochemical Characterization and Structural Insight into Interaction and Conformation Mechanisms of Serratia marcescens Lysine Decarboxylase (SmcadA)
Source: Molecules. 2021 Jan 29;26(3):697. doi: 10.3390/molecules26030697 (PMC7866039; doi:10.3390/molecules26030697)
Supplement: Supplementary file 1 [file molecules-26-00697-s001.pdf]

Biochemical characterization and structural insight into interaction and conformation mechanisms of *Serratia marcescens* lysine decarboxylase (SmcadA)

Tolbert Osire [1], Zhina Qiao[1], Taowei Yang [1*], Meijuan Xu [1], Xian Zhang [1], Zhiming Rao [1,*]

[1] The Key Laboratory of Industrial Biotechnology, Ministry of Education, School of Biotechnology, Jiangnan University, 1800 LiHu Boulevard, Wuxi, 214122, Jiangsu, China

Corresponding author

Prof. Zhiming Rao: The Key Laboratory of Industrial Biotechnology, Ministry of Education, School of Biotechnology, Jiangnan University, 1800 LiHu Boulevard, Wuxi, 214122, Jiangsu, China

Correspondence email; raozhm@jiangnan.edu.cn(Zhiming Rao)*

Supplementary data

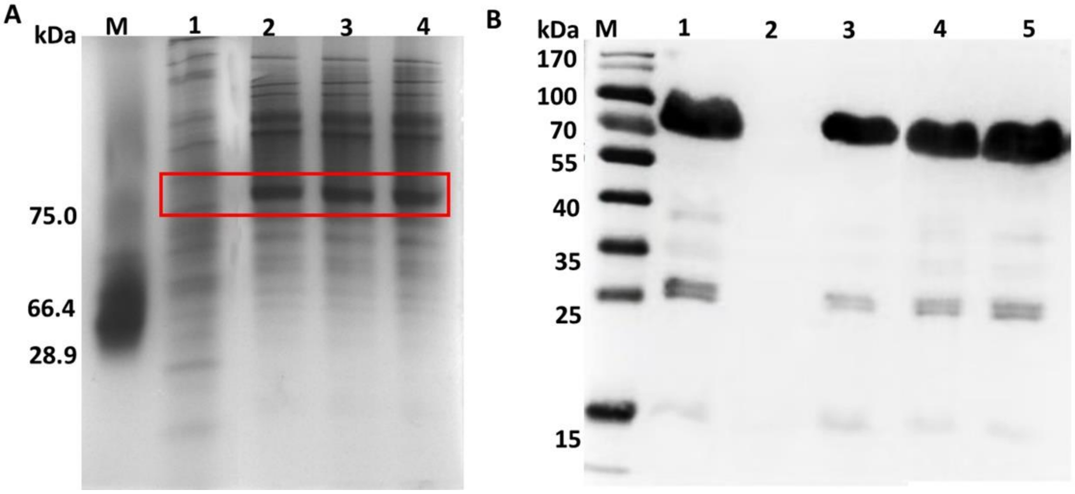

Figure S1. The native PAGE and Western blot gels of SmcadA and its variants. M. Marker band showing 28.9 kDa Proteinase K and 66.4 kDa Bovine Serine Albumin as reference markers.1 is the native PAGE of the SmcadA WT as the control. 2-4 Ser512Ala and Arg595Lys bands, respectively B. western blot gel where M. Marker is the Takara Inc. Broad protein marker; 1 represented SmcadA WT, 2; the control, 3-5 represent the Ser512Ala and Arg595Lys mutants, respectively. The experiments were run at 60 V, for 8 h, without a reference marker protein.

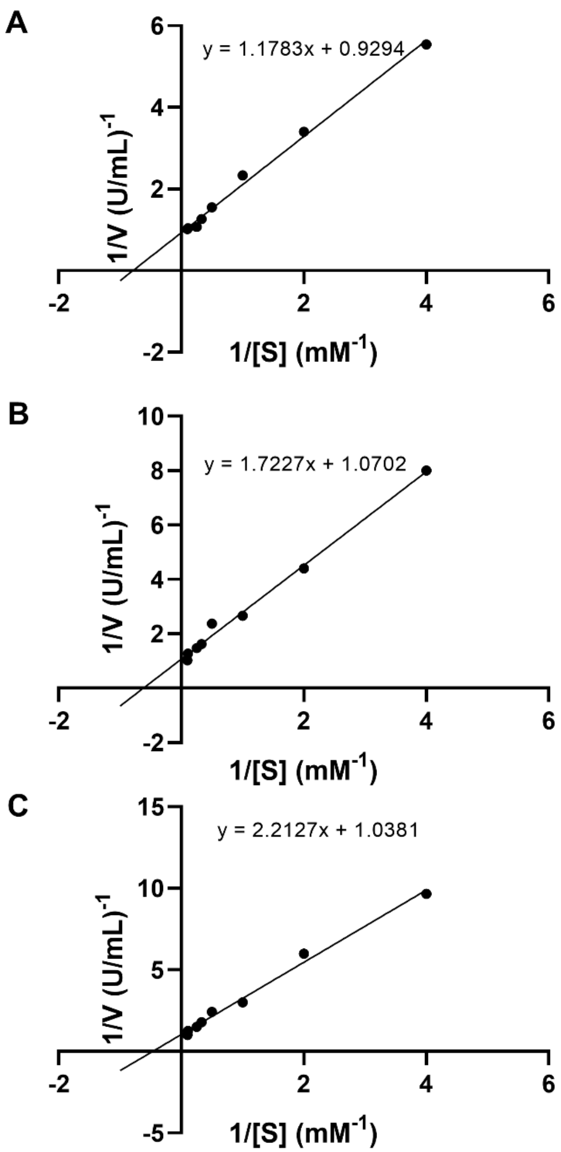

Figure S2. The Lineweaver-Burke linear plots. A-C. showed the fit for SmcadA WT, Ser512Ala and Arg595Lys mutants, respectively. The experiments were performed in a reaction mixture containing 500 mM sodium acetate buffer (350 µl), different concentrations of substrate L-lysine (5- 150 mM), 10 µl cofactor PLP (0.25 mM) for 1 hour.

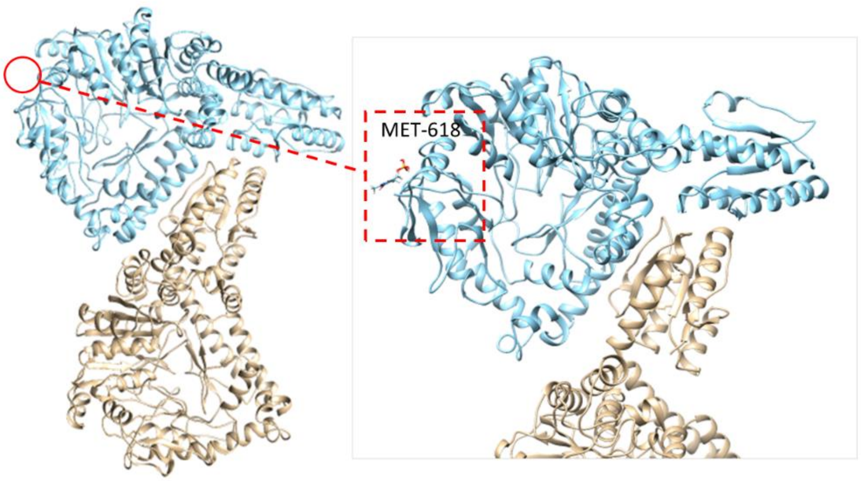

Figure S3. Image showing the first initial trajectory of cofactor PLP used during simulations.

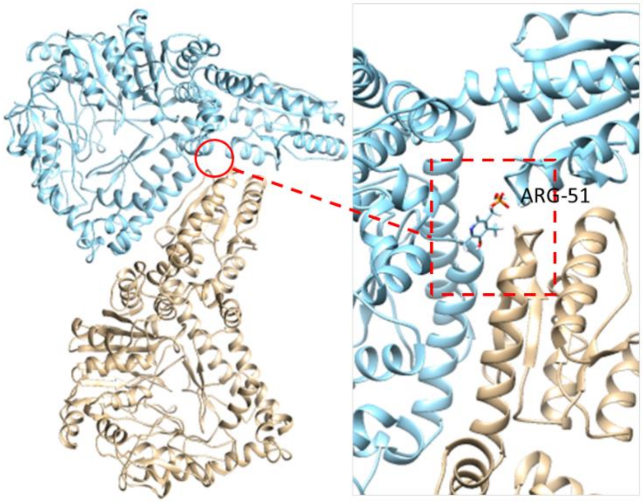

Figure S4. Image showing the second initial trajectory of cofactor PLP used during simulations.

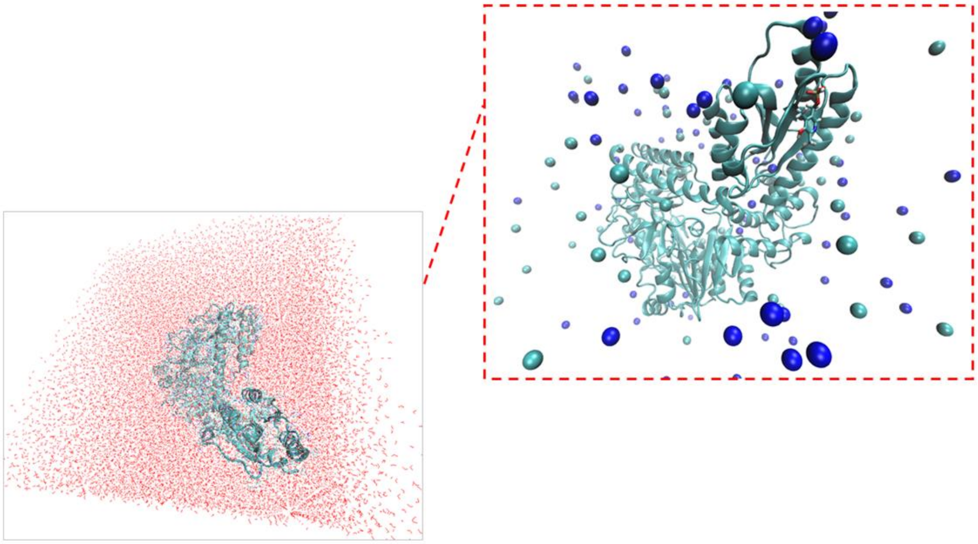

Figure S5. Solvation state of the protein-cofactor complex. The image shows the protein-cofactor complex after solvation in a triclinic dimensional box. **Inset:** The protein-cofactor after solvation and ionization. The blue spheres represent water molecules in the system, green the metal ions and the licorice sticks represent the cofactor PLP. The images were generated in VMD software.
